# Phenylethanol Glycosides Protect Myocardial Hypertrophy Induced by Abdominal Aortic Constriction via ECE-1 Demethylation Inhibition and PI3K/PKB/eNOS Pathway Enhancement

**DOI:** 10.1155/2020/2957094

**Published:** 2020-06-09

**Authors:** Qiong-Ling Fan, Jia-Wei Wang, Shi-Lei Zhang, Tao Liu, Jun Zhao, Shu-Ping You

**Affiliations:** ^1^Department of Nursing, Xinjiang Medical University, Urumqi 830011, China; ^2^Department of Public Health, Xinjiang Medical University, Urumqi 830011, China; ^3^Key Laboratory for Uighur Medicine, Institute of Materia Medica of Xinjiang, Urumqi 830004, China

## Abstract

Phenylethanol glycosides (CPhGs) are the core material basis of pharmacological activity in *Cistanche tubulosa* and have a variety of pharmacological effects. However, it is unclear whether CPhGs have an ameliorative effect on pressure overload-induced myocardial hypertrophy. In this study, male SD rats weighing (200 ± 20) g were established cardiac hypertrophy models by abdominal aortic coarctation (AAC). After operation, the rats were gavaged with corresponding medicine for 6 weeks (CPhGs 125, 250, and 500 mg/kg/d and valsartan 8.3 mg/kg/d). Echocardiography, heart weight index (HWI), cross-sectional area of cardiomyocytes (CSCA), fibrosis area, plasma endothelin 1(ET-1), and proinflammatory factors levels were detected. Our results showed that different CPhGs dosage decreased left ventricular posterior wall thickness (LVPWT), left ventricular end-diastolic diameter (LVED), HWI, CSCA, fibrosis area, ET-1, proinflammatory factors, arterial natriuretic peptide (ANP), brain natriuretic peptide (BNP), endothelin converting enzyme 1(ECE-1) mRNA levels, cyclooxygenase 2 (COX-2), high mobility group box 1 (HMGB-1) protein levels, and ECE-1 demethylation level while increasing left ventricular ejection fractions (LVEF), left ventricular fractional shortening (LVFS), phosphorylated phosphatidylinositol 3-kinase (p-PI3K), phosphorylated protein kinase B (p-PKB), and phosphorylated endothelial nitric oxide synthetase (p-eNOS). The indexes of CPhGs 250 and 500 mg/kg group were significantly different from AAC group; compared with valsartan group (AV), the indexes of CPhGs 500 mg/kg group were not significantly different. In conclusion, CPhGs ameliorated myocardial hypertrophy rats by AAC, which may be related to ECE-1 demethylation inhibition and PI3K/PKB/eNOS enhancement.

## 1. Introduction

Myocardial hypertrophy is a common adaptive response to pressure overload in hypertensive patients [1]. But with the aggravation of myocardial hypertrophy, the demand for myocardial blood supply increases, and the decrease of coronary blood flow reserve leads to myocardial injury. The development of myocardial hypertrophy is known to increase the morbidity and mortality of cardiovascular diseases [2–4]. Since hypertension is the main cause of myocardial hypertrophy, the current treatment of myocardial hypertrophy is mainly based on antihypertensive drugs, such as thiazine diuretics, calcium channel blockers, and angiotensin-converting enzyme inhibitors [5, 6]. However, the electrolyte disturbance, postural hypotension, dry cough, and other adverse reactions caused by taking these drugs remain potential risks for patients. Hence, there is an urgent need to find safer and more effective drugs.

**Figure 1:**
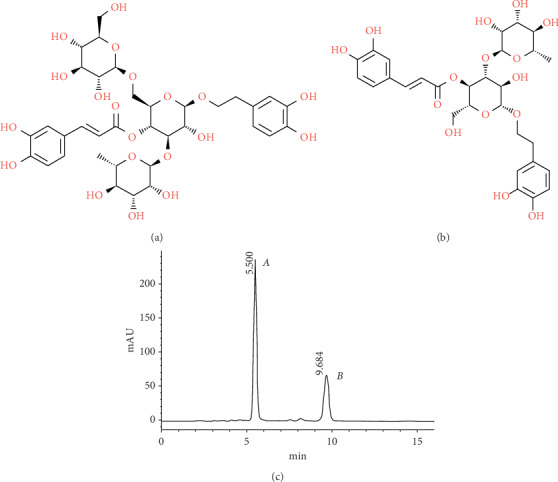
(a) Echinacoside and (b) acteoside. (c) HPLC analysis of CPhGs.

*Cistanche tubulosa*, a precious traditional Chinese herb medicine, is widely grown in Xinjiang, China. It has a variety of benefits to the consumer such as strengthening immunity, tonifying kidney impotence, improving intelligence, antioxidation, antiaging, protecting liver, and lowering blood pressure [7]. The plant contains phenylethanol glycosides (CPhGs), oleoterpenes, and polysaccharides. CPhGs are one of the effective substances contained in Cistanche. Their main components are echinosides and moleniosides (molecular structure diagrams are shown in Figure 1). [8]. In in vivo and in vitro studies, CPhGs have been proven to be effective in the preventing and treating of ischemic heart disease [9, 10]. Myocardial ischemia is also a critical pathological process of hypertrophy, but it is unclear whether the substance has a protective effect on hypertrophy.

Xinjiang Kazakh people suffer from high incidence of essential hypertension (EH) due to long-term consumption of salty milk tea [11, 12]. In the previous study, we used methylation microarray to screen the abnormal methylation sites in the blood samples of Kazakh EH patients and normal people, showing that the demethylation level of endothelin converting enzyme 1 (ECE-1) was positively correlated with EH [13]. In previous pathway analysis, it was found that ECE-1 was involved in the phosphatidylinositol 3-kinase/protein kinase B/endothelial nitric oxide synthase (PI3K/PKB/eNOS) pathway [13]. PI3K/PKB/eNOS is an important pathway in the formation of myocardial hypertrophy and the downstream pathway of ET-1 [14–16]. A prior study has shown that CPhGs liposome could inhibit the proliferation of hepatic stellate cells by regulating PI3K/PKB pathway [17]. Therefore, we hypothesised that CPhGs may play a protective role in human body by influencing ECE-1 and the PI3K/PKB/eNOS pathway.

Hence, we investigated the possible protective effect of CPhGs on myocardial hypertrophy and its effect on ECE-1 demethylation and PI3K/PKB/eNOS pathway with pressure overload myocardial hypertrophy in rats by abdominal aortic coarctation (AAC).

## 2. Materials and Methods

### 2.1. Animals

70 male SPF SD rats weighed 200 ± 20 g (10 per group) were provided by Experimental Animal Centre of Xinjiang Medical University (Urumqi, China). Animal production license number was SYXK(XIN)2016-0003 and certificate number was SCXK(XIN)2016-002. The study was approved by the Animal Ethnics Committee of the First Affiliated Hospital of Xinjiang Medical University (approval number 20180223-183). They were maintained with free access to water and food in plastic cages and kept in a 12 h light/dark cycle at a temperature of 22 ± 2°C and humidity of 45–55% in our Experimental Animal Centre.

### 2.2. Materials

CPhGs, whose content of echinacea was more than 40% and acteoside was more than 16%, were purchased from Hetian Di Chen Medical Biotechnology Co., Ltd. Valsartan was purchased from Beijing Novartis Pharma Co., Ltd. Carboxymethylcellulose sodium (CMC-Na) was provided by Xian Zhengtian Pharmaceutical Accessories Co., Ltd. And CPhGs were dissolved in 0.5% CMC-Na.

### 2.3. Abdominal Aortic Coarctation (AAC)

After anesthetized by 2.5% pentobarbital sodium (2.5 ml/kg) (Merck, Darmstadt City, Hessen, Germany), the rats were shaved and kept warm on heating pads. Then the abdominal cavity of the rat was opened for 2 cm in the middle of the abdomen in a sterile environment, the abdominal viscera were pushed to the right side, and the abdominal aorta was fully exposed at 0.5 cm above the right renal artery. Number 8 needle was ligated with number 2 wire in parallel to the abdominal cavity being sutured [18]. Abdominal aortas of rats in sham group were separated without ligation and there was no control group for the surgery. The day after the operation, AC 125 mg/kg, AC 250 mg/kg, and AC 500 mg/kg were fed with CPhGs by gavage; AV group were fed with valsartan by gavage; and control group, AAC group, and sham group were not fed with CPhGs or valsartan, once a day for 42 consecutive days. Drug dose was set on the basis of previous reference and preliminary experiment [8].

### 2.4. Echocardiography

Six weeks after AAC, the rats were anesthetized with 2.5% pentobarbital sodium and performed the echocardiography (Philips HD11 XE, Amsterdam, Netherlands). LVPWT, LVED, LVEF, and LVFS were measured four times to calculate the averages as the final indicators.

### 2.5. Heart Weight Index (HWI)

After echocardiography, the rats were executed by collecting blood from the abdominal aorta. The hearts of rats were removed from chest and weighed after being washed by normal saline (4°C) for 3 times and drained with clean filter paper. The HWI was calculated through the following formula:

(1) $$\begin{array}{c} \text{HWI}=\frac{\text{heart weight}}{\text{body weight}}. \end{array}$$

### 2.6. Hematoxylin-Eosin and Masson Staining

The myocardium from left ventricular was fixed by 4% paraformaldehyde embedded in paraffin, crosscut with a section thickness of 4 *μ*m, dried in an oven at 60°C for 2 hours, and then stained with hematoxylin-eosin or masson (Solaibio, Beijing, China). The structure of the myocardium was observed with an inverted phase contrast microscope (Leica, Frankfurt, Germany). Image *J* software was used to calculate the CSAC and fibrosis area.

### 2.7. Enzyme Linked Immune Sorbent Assay (ELISA)

Abdominal aortic blood was extracted from rats and placed in an anticoagulation tube for 2 h at room temperature and then centrifuged at 3000 r/min speed. The supernatant was absorbed with pipettes (Eppendorf, Hamburg, Germany) and stored at −80°C for testing. Plasma ET-1, interleukin 1*β*(IL-1*β*), interleukin 6 (IL-6), tumor necrosis factor *α* (TNF-*α*) (Bioss, Beijing, China), and cyclooxygenase 1 (COX-1) (Cusabio, Wuhan, China) levels of rats were detected according to ELISA kit instructions.

### 2.8. Methylation-Specific PCR

DNA was extracted from left ventricular myocardial tissue of rats by DNA extraction kit (Tiangen Biotech, Beijing, China). The absorption value (A value) was equal to OD260/OD280 (1.8∼1.9). 20 *μ*l genomic DNA (500 pg-1 *μ*g) was modified with sulfurous acid by Methylation-Gold Kit (ZYMO, Orange County, CA, USA). The MSP reaction system was 25 *μ*L, including 3.0 *μ*L of sulfite modified DNA template, 2.5 *μ*L of 10 × buffer, 2.5 *μ*L of 2.5 mm dNTPs, 1.0 *μ*L of 10 *μ*m primer, 0.1 *μ*L of hotstar Taq DNA polymerase (TAKARA Biotechnology), and 14.9 *μ*L of ddH_2_O. There were 45 cycles in total: denaturation at 95°C, annealing at 55°C, and extension at 72°C for 30 s. After PCR, 5 *μ*L product was separated by 2% agarose gel electrophoresis. The DNA was treated by M. Sssl methyltransferase (New England Biolabs, Ipswich, MA, USA) was used as the positive control of methylation, and the DNA of normal control group was used as the positive control of demethylation. Finally, the agarose gel was photographed and analyzed by Bio-Rad gel imaging system. The demethylation level was calculated through the following formula:

(2) $$\begin{array}{c} \text{Demethylation level}=\frac{\text{demethylated OD value}}{\left(\text{methylated OD value}+\text{demethylated OD value}\right)}\times 100\text{\%}. \end{array}$$

All primers designed in Methprimer (Applied Biosystems, MA, USA) and synthesized by Sangon Biotech (Shanghai, China) are listed in Table 1.

### 2.9. Quantitative Real-Time PCR

Total RNA was extracted from 50 mg left ventricular myocardial tissue by trizol (Thermo Scientific Inc., MA, US). The concentration and purity of RNA were detected by ultraviolet spectrophotometer with the standard of OD260/OD280 ratio of 1.8–2.0. After quantification, the RNA was reverse-transcribed into cDNA by RevertAid First Strand cDNA Synthesis Kit (Thermo Scientific, Inc.). The TB Green™ Premix Ex Taq™ II; were provided by Takara (Kyoto, Japan). qRT-PCR was performed by ABI QuantStudio™6 Flex Real-Time PCR system (Applied Biosystems, Foster City, CA, USA). Baseline value and threshold value were set after completion, and the circulating threshold (*C*_T_) was taken to analyze the relative expression level of the target gene by 2^−^^△△*C*^_T_ method. Primer sequences were synthesized by Sangon Biotech (Shanghai, China). All Primers used are listed in Table 2.

### 2.10. Western Blot

Proteins were extracted from left ventricular myocardium with RiPA Buffer (Thermo Scientific) and protease inhibitor was added. Protein concentration was determined by BCA method (Solarbio, Beijing, China). Proteins were taken at 50 *μ*g and denatured at 100°C for 5 mins. Proteins were separated by sodium dodecyl sulfate polyacrylamide gel electrophoresis (SDS-PAGE, Beyotime, Shanghai, China) and then transferred to a polyvinylidene fluoride (PVDF) membrane by electrophoretic equipment (Bio-Rad, CA, USA). After being blocked with 3% BSA for 2 hours at room temperature, the membranes were incubated with anti-PI3K antibody, anti-phospho-PI3K antibody, anti-eNOS antibody, anti-phospho-eNOS (1 : 1000, Bioss, Beijing, China), anti-COX-2 antibody, anti-HMGB-1 antibody (1 : 1000, *Proteintech*, Chicago, IL, USA), and anti-ECE-1 antibody (1 : 2000, *Signalway Antibody*, College Park, MD, USA), primary antibodies overnight at 4°C. Next day, after being washed with TBST for 3 times, the membranes were incubated with HRP-labeled goat anti-rabbit IgG (1 : 25000, 1 h, Zhongshanjinqiao, Shanghai, China) at room temperature. After being washed with TBST for 3 times, the membranes were visualized by Pierce™ ECL Western Blotting Substrate and imaged using FluorChem *E* Imaging System (Protein Simple, USA), which were normalized to *β*-actin as an internal control.

### 2.11. Statistical Analysis

The data were expressed as mean ± standard deviation and analyzed by one-way ANOVA test using IBM SPSS Statistics 25.0 software (Chicago, IL, USA) and GraphPad software (San Diego, CA, USA). A value of *P* < 0.05 was considered to be statistically significant.

## 3. Results

### 3.1. CPhGs Improved Cardiac Function in Rats after AAC

**Figure 2:**
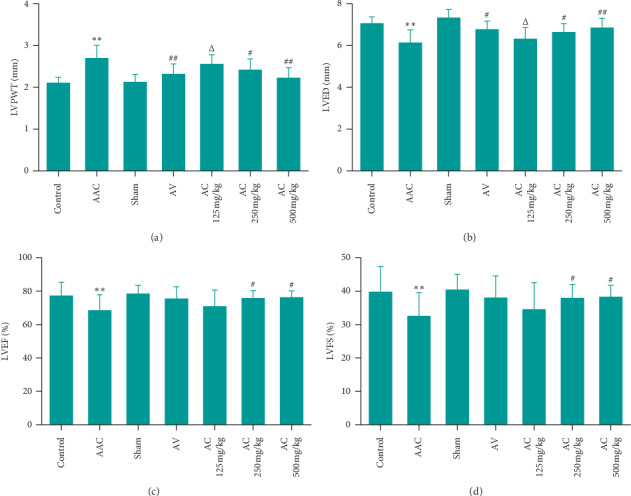
Effects of CPhGs on cardiac ultrasound parameters in rats after AAC: (a) left ventricular posterior wall thickness (LVPWT); (b) left ventricular end-diastolic diameter (LVED); (c) left ventricular ejection fractions (LVEF); and (d) left ventricular fractional shortening (LVFS). ^*∗∗*^*p* < 0.01 versus sham; ^#^*p* < 0.05, ^##^*p* < 0.01 versus AAC; ^Δ^*P* < 0.05 versus AV (*n* = 9‐10).

The rats received gavage from the day after AAC. The positive control group (AV) was given gavage with valsartan 8.3 mg/kg/d, and the CPhGs group (AC) was given gavage with CPhGs 125, 250, and 500 mg/kg/d, respectively. After six weeks' continuous dosing, the rats were performed echocardiography under 2.5% sodium pentobarbital anesthesia. Two rats died after AAC: one in model group died on the third day after AAC from pulmonary edema; another one in AC 500 mg/kg died the day after surgery suspected to be caused by surgical stress. As shown in Figure 2, compared with sham group, LVED, LVEF, and LVFS in AAC group were decreased significantly but LVPWT in AAC group was increased significantly. Compared with AAC group, LVED, LVEF, and LVFS were increased and LVPWT was decreased to varying degrees in each AC dose group; for LVPWT, significant change was not found in AC 125 mg/kg group but was found in AC 250 and 500 mg/kg groups. Compared with AV group, the LVPWT and LVED in AC 125 mg/kg group were observed significant difference, while LVPWT, LVED, LVFS, and LVFS in AC 250 and 500 mg/kg groups were not.

### 3.2. CPhGs Decreased HWI, CSAC, and Fibrosis Area in Rats after AAC

**Figure 3:**
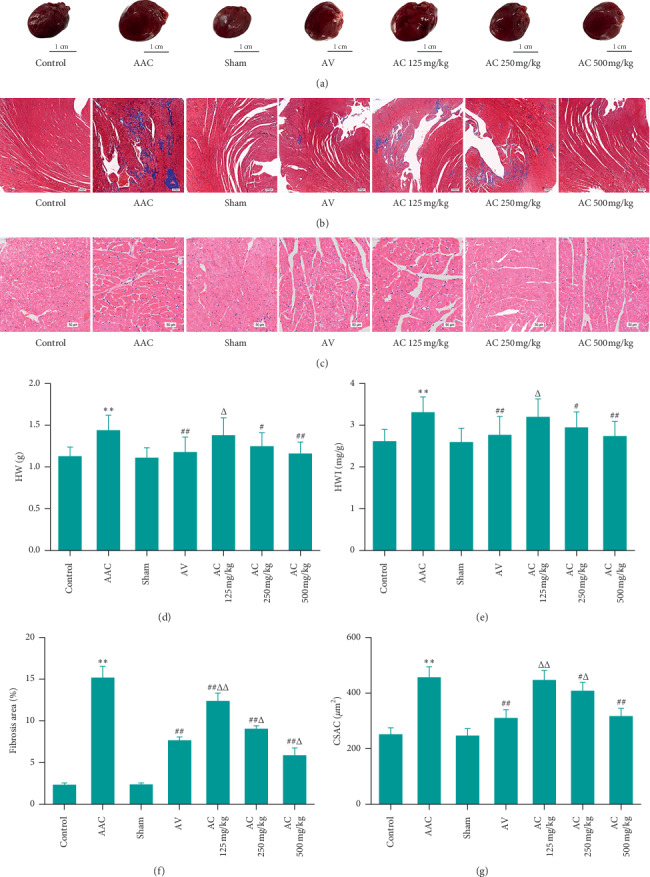
Effects of CPhGs on HWI, CSAC, and fibrosis area in rats after AAC. (a) Heart shape, bar = 1 cm; (b) masson staining of rat myocardium, bar = 200 *μ*m; and (c) HE staining of rat myocardium, bar = 50 *μ*m; (d–g) heart weight (HW), heart weight index (HWI), fibrosis area, and cross-sectional area of cardiomyocytes (CSAC). ^*∗∗*^*P* < 0.01 versus sham; ^#^*P* < 0.05, ^##^*P* < 0.01 versus AAC; ^Δ^*P* < 0.05, ^ΔΔ^*P* < 0.01 versus AV (*n* = 9‐10).

Then we weighed the heart of each rat to calculate the HWI and took the myocardial tissue for HE and masson staining to observe the changes of the cross-sectional area (CSAC) and fibrosis area in cardiomyocytes. As shown in Figures 3(a), 3(d), and 3(e), compared with sham group, the heart weight (HW) and HWI in AAC group were reduced significantly; compared with AAC group, HW and HWI in AC250 and 500 mg/kg groups were reduced significantly; compared with AV group, HW and HWI were increased significantly in AC 125 mg/kg group but were not in AC 250 and 500 mg/kg groups. In Figures 3(b) and 3(f), compared with sham group, the masson staining results showed that the fibrosis area in AAC group was increased significantly. For fibrosis area, significant decreasing was found in AC 125, 250, and 500 mg/kg groups when compared to AAC group, while significant increasing in AC 125 and 250 mg/kg groups and significant decreasing in AC 500 mg/kg group were found when compared to AV group. In Figures 3(c) and 3(g), the HE staining results showed that CSAC in AAC group was increased significantly in comparison with that in the sham group; compared with AAC group, CSAC in AC 250 and 500 mg/kg groups was reduced significantly; compared with that in AV group, however, CSAC was increased significantly in AC 125 and 250 mg/kg groups, and no significant difference was found in the AC 500 mg/kg group.

### 3.3. CPhGs Reduced mRNA Levels of Myocardial Hypertrophy Genes in Myocardial Tissue of Rats after AAC

**Figure 4:**
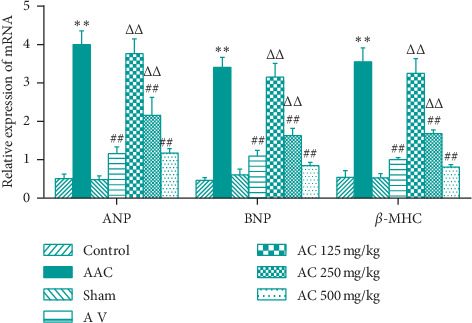
Effect of CPhGs on the expression of myocardial hypertrophy gene mRNA in myocardial tissue of rats after AAC. ^*∗∗*^*P* < 0.01 versus sham; ^##^*P* < 0.01 versus AAC; ^ΔΔ^*P* < 0.01 versus AV (*n* = 9-10).

ANP, BNP, and myosin heavy chain *β* (*β*-MHC) are important cardiac hypertrophy factors [19, 20]. We detected the relative expression levels of these three cardiac hypertrophy genes in the myocardial tissues. As shown in Figure 4, compared with sham group, the relative expression levels of ANP, BNP, and *β*-MHC mRNA in AAC group were increased significantly; however, compared with AAC group, the relative expression levels of the three myocardial hypertrophy genes in AC 250 and 500 mg/kg groups were decreased significantly. The expression levels in AC 500 group were not significantly different compared to those in AV group.

### 3.4. CPhGs Reduced the Proinflammatory Factors in Plasma of Rats after AAC

**Figure 5:**
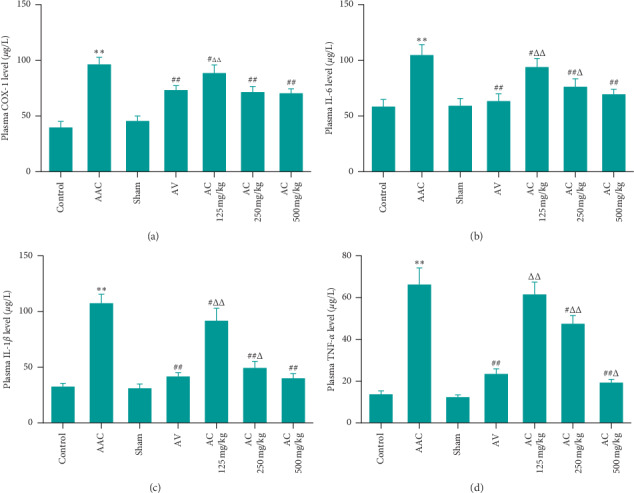
Effect of CPhGs on proinflammatory factor levels in plasma of rats after AAC. (a) Cyclooxygenase 1 (COX-1); (b) interleukin 6 (IL-6); (c) interleukin 1*β* (IL-1*β*); and (d) tumor necrosis factor *α* (TNF-*α*). ^*∗∗*^*P* < 0.01 versus sham. ^*∗∗*^*P* < 0.01 versus sham. ^#^*P* < 0.01, ^##^*P* < 0.01 versus AAC. ^Δ^*P* < 0.01, ^ΔΔ^*P* < 0.01 versus AV (*n* = 9-10).

Plasma levels of proinflammatory factors such as COX-1, IL-6, IL-1, and TNF-*α* of rats in each group were determined. As shown in Figure 5, compared with sham group, the four proinflammatory factors in AAC group were significantly increased. Compared with the AAC group, the proinflammatory factors in each CPhGs dose group decreased significantly, except for TNF-*α* (5D) of AC125 mg/kg groups. Compared with the AV group, the levels of COX-1, IL-6, and IL-1*β* in plasma of the AC 500 mg/kg group showed no significant difference (5ABC); however the levels of TNF-*α* in plasma of the AC 500 mg/kg group were lower than those in the AV group. Therefore, CPhGs may have a better effect on reducing proinflammatory factors in rats with cardiac hypertrophy after AAC surgery.

### 3.5. CPhGs Reduced the Expression of Inflammatory Proteins in Myocardial Tissue of Rats after AAC

**Figure 6:**
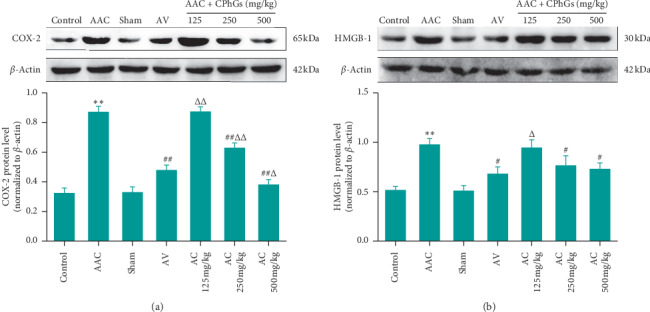
Effects of CPhGs on the expression of COX‐2 and HMGB‐1 proteins in myocardial tissues of rats after AAC. (a) Cyclooxygenase 2 (COX‐2) and (b) high mobility group protein B1 (HMGB‐1). ^*∗∗*^*P* < 0.01 versus sham. ^#^*P* < 0.05. ^##^*P* < 0.01 versus AAC. ^Δ^*P* < 0.05, ^ΔΔ^*P* < 0.01 versus AV (*n* = 6).

The inflammatory response also plays an important role in the pathological process of myocardial hypertrophy [21, 22]. We detected the relative expression levels of cyclooxygenase 2 (COX-2) and high mobility group protein B1 (HMGB-1) in rat myocardial tissues by western blot. As shown in Figure 6, compared with sham group, the relative expression levels of COX-2 and HMGB-1 protein group were increased significantly in AAC; meanwhile, compared with AAC group, they were reduced significantly in AC 250 and 500 mg/kg groups. For the relative protein expression level of COX-2, it was significantly higher in AC 125 and 250 mg/kg groups but significantly lower in AC 500 mg/kg group than that in AV group. For the relative expression level of HMGB1 protein, compared with AV group, it presented a significantly upward tendency in AC 125 mg/kg group and no significant difference in AC 250 and 500 mg/kg groups was found.

### 3.6. CPhGs Reduced the Demethylation Level of ECE-1 in Myocardial Tissue of Rats after AAC

**Figure 7:**
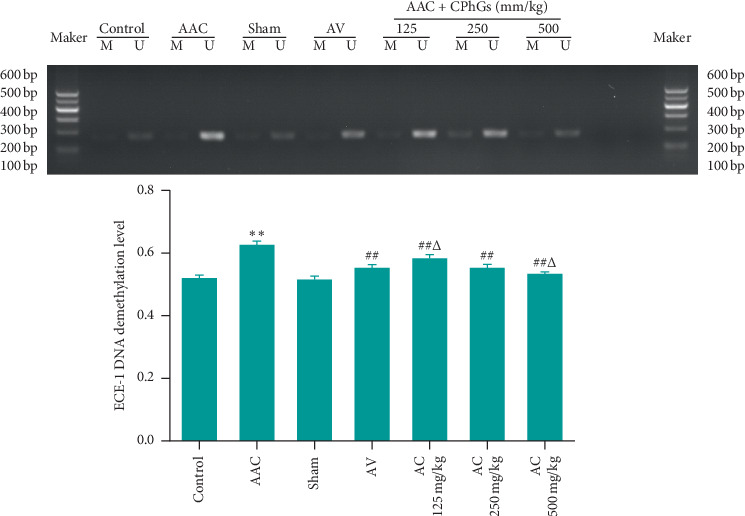
Effect of CPhGs on the demethylation level of ECE-1 gene in myocardial tissue of rats after AAC. M: means methylation level, U: means demethylation level. ^*∗∗*^*P* < 0.01 versus sham, ^#^*P* < 0.05, ^##^*P* < 0.01 versus AAC, and ^Δ^*P* < 0.05 versus AV (*n* = 6).

In previous study, we found that ECE-1 demethylation worked on hypertension and might have effect on the production of ET-1 [13]. Accordingly, we wondered whether CPhGs would affect the demethylation level of ECE-1. As shown in Figure 7, compared with sham group, the methylation level of the ECE-1 in the myocardial tissue was increased significantly in the AAC group. At the same time, it was reduced significantly in AC 125, 250, and 500 mg/kg groups compared with AAC group. The level of ECE-1 gene demethylation in AC 125 mg/kg group was significantly higher than that in AV group; there was no significant difference in AC 125 mg/kg group, but a significant drop was found in AC 500 mg/kg group when compared to AV group.

### 3.7. CPhGs Decreased the Expression of ECE-1 in Myocardial Tissue of Rats after AAC

**Figure 8:**
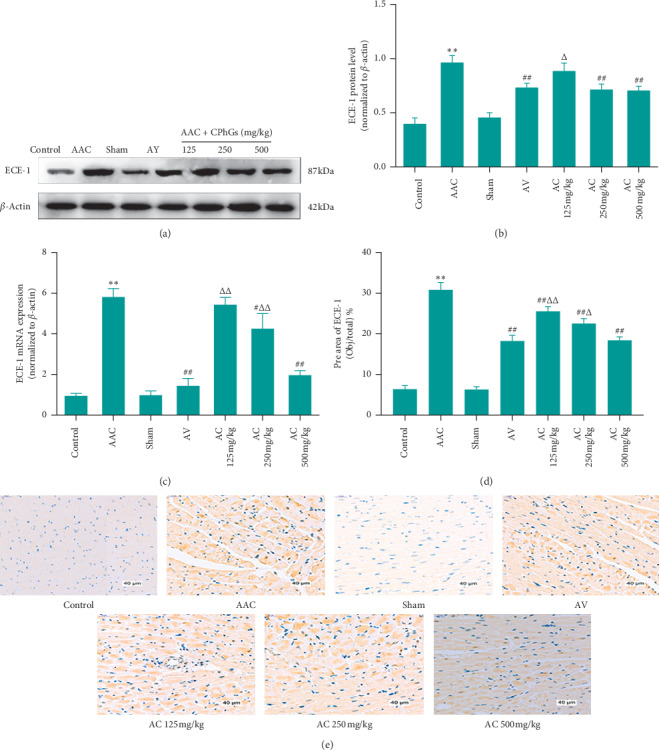
Effect of CPhGs on the expression of ECE-1 in myocardial tissue of rats after AAC. (a), (b) ECE-1 protein level; (c) ECE-1 mRNA expression; and (d), (e) immunohistochemistry of ECE-1 in myocardial tissue. Bar = 20 *μ*m. ^*∗∗*^*P* < 0.01 versus sham;^#^*P* < 0.05, ^##^*P* < 0.01 versus AAC; ^Δ^*P* < 0.05, ^ΔΔ^*P* < 0.01 versus AV. (*n* = 6).

To further understand the effect of ECE-1 methylation, we detected the expression of mRNA, protein, and immunohistochemistry of ECE-1 in myocardial tissue. As shown in Figure 8, compared with sham group, the relative expression levels of ECE-1 mRNA, relative expression levels of protein, and the average area of myocardial immunohistochemistry were reduced significantly in AAC group.  Figure 8(c) reveals that, compared with the AAC group, the relative expression levels of ECE-1 mRNA were reduced significantly in AC 250 and 500 mg/kg groups; when compared to the AV group, they were significantly higher in AC 125 and 250 mg/kg groups but were not significantly different in AC 500 mg/kg group. Meanwhile, as can be seen in Figures 8(a) and 8(b), compared with AAC group, relative expression levels of ECE-1 protein were significantly decreased in the AC 250 and 500 mg/kg group; however, they were not significantly different in the AC 250 and 500 mg/kg group compared to those in AV group. Figures 8(d) and 8(e) indicate that, compared with AAC group, the expression area of ECE-1 in the myocardial tissue of rats was reduced significantly in AC 125, 250, and 500 mg/kg group; however, compared with AV group, it was significantly higher in AC 125 and 250 mg/kg groups and showed no significant difference in AC500 mg/kg groups.

### 3.8. CPhGs Reduced the Plasma ET-1 Level in Rats after AAC

**Figure 9:**
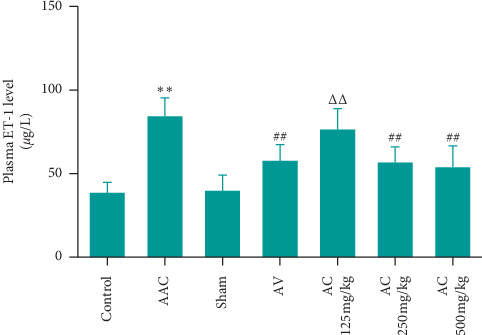
Effect of CPhGs on plasma ET-1 levels in rats after AAC. ^*∗∗*^*P* < 0.01 versus sham, ^##^*P* < 0.01 versus AAC, ^ΔΔ^*P* < 0.01 versus AV (*n* = 9‐10).

We also measured plasma ET-1 levels in rat by ELISA, as shown in Figure 9, compared with sham group, the plasma levels of ET-1 in AAC group were increased significantly. Meanwhile, the plasma levels of ET-1 showed ascending trend in AC 125 mg/kg group but indicated no significant difference in AC 250 and 500 mg/kg groups when compared to those in AV group.

### 3.9. CPhGs Increased the Expression of PI3K/PKB/eNOS Pathway in Rats after AAC

**Figure 10:**
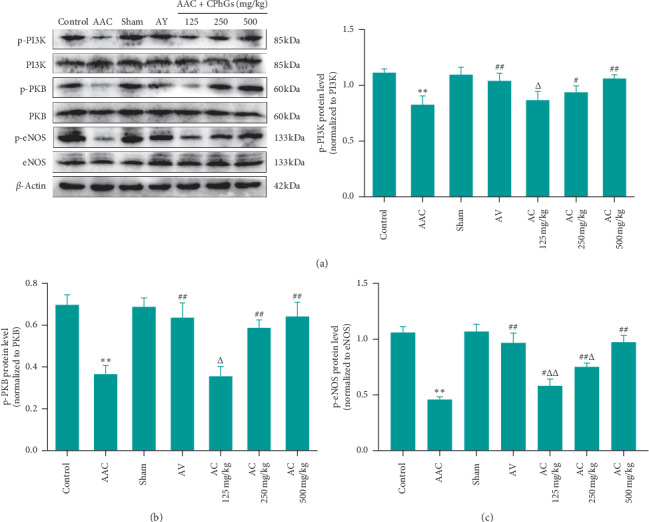
Effect of CPhGs on the expression of PI3K/PKB/eNOS pathway in myocardial tissue of rats after AAC. (a) p-PI3K protein level; (b) p-PKB protein level; and (c) p-eNOS protein level. ^*∗∗*^*P* < 0.01 versus sham, ^#^*P* < 0.05, ^##^*P* < 0.01 versus AAC, ^Δ^*P* < 0.05 versus AV. (*n* = 6).

PI3K/PKB/eNOS is an important pathway in the process of myocardial hypertrophy and has an important connection with ET-1 [14–16]. Therefore, we further detected the proteins expression levels of PI3K/PKB/eNOS pathway in rat myocardial tissue. As shown in Figure 10, compared with sham group, the relative expression levels of p-PI3K, p-PKB, and p-eNOS proteins in AAC group were reduced significantly; however compared with AAC group, the relative expression levels of p-PI3K were increased significantly in AC 250 and 500 mg/kg groups. At the same time, the relative expression levels showed a descending trend in AC 125 mg/kg group and showed no significant difference in AC 250 and 500 mg/kg groups compared to those in AV group. As shown in Figure 10(b), compared with AAC group, the relative expression levels of p-PKB were increased significantly in AC 250 and 500 mg/kg group. Meanwhile, they were significantly lower in AC 125 mg/kg group and were not significantly different in AC 250 and 500 mg/kg groups compared to those in AV group. As shown in Figure 10(c), compared with AAC group, the relative expression levels of p-eNOS were increased significantly in AC 125, 250, and 500 mg/kg groups; however, compared with AV group, the relative expression levels presented a significantly downward trend in AC 125 and 250 mg/kg groups but showed no significant difference in AC 500 mg/kg group.

## 4. Discussion

Compared with hypertension patients, those with hypertension and cardiac hypertrophy have a 6-8 times increased probability of acute myocardial infarction, chronic heart failure, and even sudden cardiac death, which seriously affects the treatment and prognosis of hypertension [23, 24]. Under such circumstance, the treatment of hypertension should not be limited to the control of blood pressure. Accordingly, the reversal of cardiac hypertrophy, as the key factor, also deserves great concern. In patients with cardiac hypertrophy, cardiomyocyte protein synthesis is increased in number, enlarged in size, thickened in cell walls, together with the occurrence of more sarcomere, interstitial fibroblast proliferation, and cardiac collagen proliferation. At the same time, the expression of cardiac hypertrophy markers, such as ANP, BNP, and *β*-MHC mRNA, is also increased [25–27].

As a traditional Chinese medicine for the treatment of female infertility and male impotence, the stem of *Cistanche tubulosa* was first recorded in the Shen Nong's Materia Medica in ca. 100 B.C. and is currently widely used as a kind of healthy food in the Southeast Asia area [28]. In recent years, *Cistanche tubulosa* has drawn the attention of the medical community because of its significant biological activity. As a traditional antifatigue herb, not only *Cistanche tubulosa* has the function of muscle protection, but also its extract can improve ATP storage and reduce muscle damage after exercise in rats [29]. According to the results of ex vivo experiments, *Cistanche tubulosa* also has a protective effect against statin-induced muscle toxicity with the help of caspase pathway [30]. In addition, researchers have studied the pharmacological effects of *Cistanche tubulosa* a in protecting cardiovascular and other aspects and found that *Cistanche tubulosa* could inhibit the increase of fasting blood glucose and postprandial blood glucose, improve insulin resistance, and ameliorate dyslipidemia, as well as inhibiting weight loss in db/db mice [31]. Previous studies showed that CPhGs can reduce the damage of free radicals to myocardial mitochondrial membrane and plasma omentum, reduce malondialdehyde content, reduce myocardial ultrastructural damage, increase myocardial mitochondrial antioxidant enzyme activity, reduce myocardial infarct size, improve the activity of phosphocreatine in myocardial tissue, and have a protective effect on ischemic myocardium [9, 10, 32]. We aimed to explore the effects of CPhGs on pressure overload-induced cardiac hypertrophy. In this study, it was found that CPhGs decreased LVPWT, LVED, HWI, AMC, and cardiac hypertrophy gene levels (i.e., ANP, BNP, and *β*-MHC), increased EF and FS, inhibited cardiac hypertrophy, and improved cardiac function in rats. Besides, inflammation is one of the key phenotypes in the pathogenesis of cardiac hypertrophy [33]. Some scholars had found that deletion of IL-6 might attenuate pressure overload-induced left ventricular hypertrophy and dysfunction [34]. And we found that CPhGs could not only decrease the plasma IL-6 level of rats after AAC, but also reduce other proinflammatory, such as COX-1, IL-1*β*, and TNF-*α*. In addition, CPhGs can also reduce COX-2 and HMGB-1 protein related to inflammation. The effects of CPhGs were dose-dependent with increasing drug doses. Moreover, it is worth mentioning that the high dose of CPhGs group had a significant protective effect on hypertrophic rats, similar to the effect of valsartan positive group. Therefore, CPhGs could be a potential pharmacological agent to inhibit pressure overload-induced cardiac hypertrophy.

DNA methylation refers to a molecular DNA methylation modification process in which S-adenosyl-L-methionine acts as a methyl donor to obtain a methyl group through covalent bonding under the catalysis of DNA methyltransferases [35]. It is well known that ET-1 is the most potent vasoconstrictor and can strongly constrict medullary and medullary arteries, resulting in decreased natriuresis and increased blood pressure [36–38]. Being the key enzyme in the final step of endothelin production, ECE-1 not only coexists with ET-1 in cardiovascular diseases, but also can regulate ET-1 production. Thus, ECE-1 can be rather essential to the development and progression of the disease [39]. In in vitro experiments on vascular endothelial cells, methylation of the CpG island in the ECE-1c promoter region decreases its transcriptional activity, resulting in decreased expression of ECE-1c, further leading to decreased production of ET-1 and decreased blood pressure in vivo, and this line of changes suggests that ECE-1 methylation may be involved in the pathology of hypertension [40, 41]. In our previous study, we also have found increased demethylation of ECE-1 in hypertensive patients [35]. The results of the present study showed that CPhGs significantly reduced ECE-1 demethylation, decreased ECE-1 mRNA and protein expression, and reduced plasma ET-1 levels in the myocardium of rats with pressure overload-induced cardiac hypertrophy after AAC.

Whether CPhGs affect ECE-1 demethylation levels and whether they protect against cardiac hypertrophy in rats have not been clarified. PI3K is the final pathway for cardiomyocyte hypertrophy from contractile to hypertrophic type [42]. Both isoforms of PI3K can be involved in cardiomyocyte hypertrophy, in which Pll0*α* is involved in physiological hypertrophy, while p110*γ* is involved in pathological hypertrophy. P110, when knocked out in mice, can protect against cardiomyopathic pathological stimuli and plays a crucial role in the development and progression of cardiomyocyte hypertrophy [43]. PKB is central to this signaling pathway. Its activation can modulate molecular function by activating or inhibiting downstream effector molecules through phosphorylation [44]. PI3K/PKB is also a key signaling pathway behind ET-1, and studies have shown that ET-1 can independently induce fibroblast resistance to apoptosis through PI3K/PKB signaling activation [45]. Other studies have shown that ET-1 can regulate the activity of eNOS and the release of NO through endothelin receptor B1 [46]. As an important vasodilator, NO can antagonize the vasoconstrictive effects of ET-1 from multiple levels and maintain normal cardiovascular function [47]. In this study, the middle and high dose CPhGs groups showed a significant increase in p-PI3K, p-PKB, and p-ENOS. However, the high dose CPhGs group had the same effect as AV group, which further demonstrated that CPhGs could inhibit cardiac hypertrophy in rats after AAC by activating the PI3K/PKB/eNOS signaling pathway. Other studies have also found a link between the PI3K/PKB pathway and inflammatory responses [48, 49]. Zou and other researchers have reported that in asthma, Brahman-related genes play an essential role in maintaining airway inflammation and affecting the PI3K/Akt/mTOR pathway [50]. In addition, CPhGs also decreased the expression of inflammation-related genes in myocardial tissue. Therefore, we speculated that CPhGs may attenuate the inflammatory response in stress-overloaded rats by increasing the PI3K/PKB/eNOS signaling pathway.

Overall, our study is the first to demonstrate the protective effect of CPhGs on pressure overload-induced cardiac hypertrophy in rats after AAC, which may be related to the role CPhGs play in effectively reducing the demethylation level of ECE-1 and enhancing the PI3K/PKB pathway. This finding provides a new idea for the clinical research of hypertension hypertrophy and the development of new therapeutic drugs to prevent, control, and reverse hypertension hypertrophy. Although there are still many key questions waiting to be addressed, further understanding of the mechanisms by which CPhGs and PI3K/PKB mediate inflammatory responses may shed new light on exploring cardiac hypertrophy treatment in the future.

## 5. Conclusions

Our results showed that CPhGs ameliorated myocardial hypertrophy rats by AAC, which may be related to ECE-1 demethylation inhibition and PI3K/PKB/eNOS enhancement.

## Figures and Tables

**Table 1:** The primer sequences of ECE-1 used for methylation-specific PCR.

Gene	Forward primer (5′-3′)	Reverse primer (3′–5′)
Methylation primer (M)	GGCGTTAGGTAGGAGATAAAGGTC	TCTAACTACCCCGAATACCGATAA
Demethylation primer (U)	GTAGGTGTTAGGTAGGAGATAAAGGTTG	ACTCTAACTACCCCAAATACCAATAAA

**Table 2:** The primer sequences used for real-time PCR assay.

Genes	Forward primer (5′-3′)	Reverse primer (3′–5′)
ANP	GAGAGCGGACTAGGCTGCAA	TCAGTGGCAAlGCGACCAA
BNP	CGGATTGGCGCAGTCAGTCG	AGAGCCGCAGGCAGAGTCAG
*β*-MHC	GTGCCAATGACGACCTGAAGGAG	CTGGTTGATGAGGCTGGTGTTCTG
ECE-1	TCATCGGCTCACTCTCCAACTCC	CCTTCTTCCACCTGTGTTCTCTGC
*β*-Actin	TGTCACCAACTGGGAGGATA	GGGGTGTTGAAGGTCTCAAA

RNA expression levels were measured by SYBR on 7500 Real-Time PCR System (Applied Biosystems, USA). Each experiment was conducted in triplicate.

## Data Availability

All data included in this study are available upon request by contact with the corresponding author.
